# Learning in virtual space: an intergenerational pilot project

**DOI:** 10.3205/zma001433

**Published:** 2021-02-15

**Authors:** Claudia Schlegel, Alain Geering, Uwe Weber

**Affiliations:** 1 Berner Bildungszentrum Pflege, Bern, Switzerland

**Keywords:** virtual reality, generations, learning

## Abstract

**Introduction:** More and more educational institutions are discovering the advantages of settings for digital teaching and learning and the technology of virtual reality (VR). This also holds true for the BZ Pflege in the field of continuing education with age-heterogeneous groups of participants. The question arises whether baby-boomers and X and Y generation learners accept, understand and perceive the digital form of learning with VR glasses as beneficial for their learning.

**Project description:** A course has been developed with the goal of teaching the anatomy of the heart by immersive visualisation. A questionnaire was used to determine how the use of VR glasses influenced participants` learning, acceptance, understanding and cognitive load.

**Results: **The participants reacted positively to the VR technology. The new learning technology did not lead to cognitive overload. Participants indicated that they were able to link new knowledge with already existing knowledge. They also found the VR glasses easy to use.

**Conclusion: **From the perspective of participants and project management alike, it can be said that age- heterogeneous groups present no obstacle for new innovative teaching methods, such as the use of VR glasses.

## Zusammenfassung

**Einleitung: **Immer mehr Bildungsinstitutionen entdecken die Vorteile digitaler Lehr- und Lernsettings und auch der Virtual-Reality-Technologie (VR). So auch das Berner Bildungszentrum Pflege (BZ Pflege) im Bereich der Weiterbildung mit altersheterogenen Gruppen von Teilnehmenden. Es stellt sich die Frage, ob Lernende, die den Generationen Babyboomer, X und Y angehören, die digitale Lernform mit der VR-Brille akzeptieren, verstehen und als förderlich für ihr Lernen wahrnehmen.

**Projektbeschreibung: **Die Verantwortlichen haben eine Lehrveranstaltung entwickelt mit dem Ziel, die immersive Visualisierung der Herz-Anatomie zu unterrichten. Anhand eines Fragebogens wurde erhoben, welchen Einfluss der Einsatz der VR-Brille auf das Lernen, die Akzeptanz, die Verständlichkeit und die kognitive Belastung der Teilnehmenden hat.

**Ergebnisse: **Die Teilnehmenden haben auf den Einsatz mit der VR Technologie zustimmend reagiert. Die neue Lerntechnologie führte nicht zur kognitiven Überlastung. Die Teilnehmenden geben an, dass sie neues Wissen mit bereits vorhandenem verknüpfen konnten. Des Weiteren empfanden sie den Umgang mit der VR-Brille als verständlich.

**Schlussfolgerung:** Aus Sicht der Teilnehmenden und der Projektleitung kann gesagt werden, daß altersheterogene Gruppen für neue innovative Lehrmethoden, wie beispielsweise den Einsatz der VR-Brille, kein Hindernis darstellen.

## Introduction

Learning is becoming increasingly digital: More and more educational institutions are discovering the advantages of digital teaching and learning settings, including virtual reality technology [1]. This also applies to the training and continuing education of healthcare professionals. The term “virtual reality” means the simultaneous display and perception of a real-time, computer-generated, interactive, virtual environment [1]. 

When we put on VR glasses, we are intentionally immersing ourselves in another reality. We see an artificially-created environment and give ourselves over to this illusion. The experience in this artificial world seems so real to us that it temporarily becomes our reality [2] According to Burdea and Coiffet [3], the following three “i’s” are necessary for learning with virtual realities: immersion, imagination and interaction. In the realm of virtual learning, immersion means immersion in a scene with an artificially-created environment. The real world is simulated so that learning can take place while engaging as many senses as possible. Constant interaction with the learning content – but also with other learners, subjects and contexts – is crucial for the immersive experience. Learning often occurs with realistic input devices within the simulated environment. Imagination stands for the mental picture learners put themselves into in the virtual environment. Real-time audio-visual feedback allows the learner to constantly interact in the virtual world during the action [4].

Using VR glasses in lectures in a context-related way adds didactic value. This method supplements the lessons in a meaningful way. At the BZ Pflege, VR glasses are being integrated into the lessons as a pilot project. 

The Department of Continuing Education of the BZ Pflege offer various postgraduate studies for qualified nursing professionals, for example, in the fields of anaesthesia, emergency and intensive care. The continuing education programmes are aimed at qualified nurses of all ages. The trend towards lifelong learning means that students from different generations benefit from postgraduate studies: Both baby boomers and members of the X, Y and Z generations are represented.

A generation is a group of people who share a time-related similarity due to a common imprint of historical or cultural experiences [5]. In their youth, digitisation was not an issue for baby boomers, who were born between 1946 and 1964. Generation X, born between 1965 and 1980, are called “digital immigrants” because they did not learn to use computers until adulthood. Generation Y, born between 1981 and 2000, are called “Digital Natives” – they were born into the digital age. Generation Z, the youngest in the sequence, are marked by an era in which technology dominates everyday life. Generation Z communicates and interacts via the Internet - whether privately or professionally [5].

The age-heterogeneity of the target group poses a challenge for teachers, as they believe that VR technology is more suited to young learners. However, the goal of further education is to address all age groups didactically.

Therefore, it makes sense to apply a pedagogically-balanced mix of teaching methods. Blended learning, also known as integrated learning, is a concept that has been used at universities and technical colleges for quite some time. It is a form of learning that aims at a didactically meaningful combination – or blend – of traditional classroom instruction and modern e-learning. Blended learning uses the possibilities of networking available today via the Internet or Intranet and combines them with classical learning methods and media to create an optimal learning arrangement. It enables learning, communication, information and knowledge management independent of time and place.

At the same time, these elements are combined with experience sharing, role play and personal encounters in classical classroom training [6]. Blended learning is also used in postgraduate studies at the BZ Pflege. 

Supplementing a sequence teaching the anatomy of the heart to an age-heterogeneous group by using VR technology is seen as a didactically exciting challenge. The teachers do not know how the members of the different generations, and especially older participants, will respond to it. Even retirement and nursing homes use VR glasses for biographical memory work, for example, or for a calming effect. These applications are predominantly accepted by the residents [7]. Nevertheless, little is known about how learners who are baby boomers or who belong to Generation X react to it in education and training. Huang et al. [8], therefore, state in their study that evaluating the acceptance of such virtual learning environments is crucial. This will ensure that VR technologies are used as effectively as possible. In this context, the questions arise whether the new digital learning form, with the use of VR glasses, will be accepted by age-heterogeneous groups, consisting of baby boomers and members of Generations X and Y. In addition, it will be investigated whether the participants perceive a cognitive burden through VR glasses and whether this form of learning is perceived as understandable and conducive to learning.

## Project description

### Research design

In order to evaluate whether learners accept the new digital form of learning and perceive it as understandable and beneficial to their learning, the “One-Shot Study Design” was chosen [9], which consists of an intervention and a survey of the learners.

#### Participants

At the BZ Pflege, 32 certified nursing professionals participated in the VR pilot course (23 female, 9 male). 1 student (female) is a baby boomer, while 8 belong to Generation X (female) and 23 to Generation Y (male and female). Their average age is 31.78 years.

#### Description of the course

The course was carried out as a teaching project at the BZ Pflege in the Department of Further Education within the postgraduate studies of Anaesthesia, Intensive Care and Emergency Care. The aim was to enable the participants to experience the immersive visualisation of the anatomy of the heart and, thus, to expand their knowledge. A timeframe of 100 minutes was made available and the VR-setting took place as group work. A training room with beamer, VR headset (VR glasses) and a controller were available for the event. The participants formed small groups of three to five persons. Each group member received a task card containing the technical assignment with questions on the anatomy of the heart. There were assignments on the following topics: anatomical position of the heart and the main blood vessels, anatomical position and function of the heart valves, blood flow through the heart, anatomical position of the coronary vessels, anatomical position of the elements of the cardiac conduction system. Each group member had to show and explain the required elements of cardiac anatomy based on their task. The group could follow the first-person-view of the group member with the VR-headset via a beamer projection. Together, the participants of the group discussed the assignment and exchanged their existing knowledge. The solution on the back of the task card was used to check the group's results. An expert gave brief instructions on how to handle and navigate by means of the controller.

By using VR, course participants experience an immersive visualisation of the anatomy of the heart and can build on their previous knowledge by acting and exploring. In addition, the participants` personal knowledge is expanded through a collaborative process with the group and transferred from implicit to explicit, as facts are addressed, exchanged and made visible.

#### Evaluation

Immediately after the intervention with VR glasses, the participants were asked to fill out a questionnaire about their experience with VR technology to find out whether they perceived the new form of digital learning as beneficial. The evaluation questions were based on the questionnaire by Reif et al. *Fragegebogen zur Einschätzung ihrer Tätigkeit* *in der virtuellen Kommissionierumgebung* (Questionnaire to assess your activity in the virtual order-processing environment) [10], which is divided into three main aspects: cognitive load, motivation and acceptance and 3D visualisation (understandability).

The aspect of cognitive load is covered by three questions in the questionnaire; by the question on subjective perception to increase one's own learning, on linking new knowledge with already existing knowledge and on the feeling of having successfully mastered the task set [11]. The aspect of acceptance is defined as the characteristic of an innovation by which attempts are made to achieve positive reactions of the persons concerned when it is introduced [12]. This is covered by the questions whether or not participants liked the learning technology and if they would recommend it. 

The questionnaire`s individual criteria were modified to make sense in the context of the investigation. For example, the term “order-processing” was disregarded, since the teaching is about the anatomy of the heart and not about order-picking.

## Results

All 32 participants filled out the questionnaire. In order to guarantee anonymity, nobody was asked to give their age on the questionnaire, since only one person is a baby boomer.

### Cognitive load

**Figure 1:**
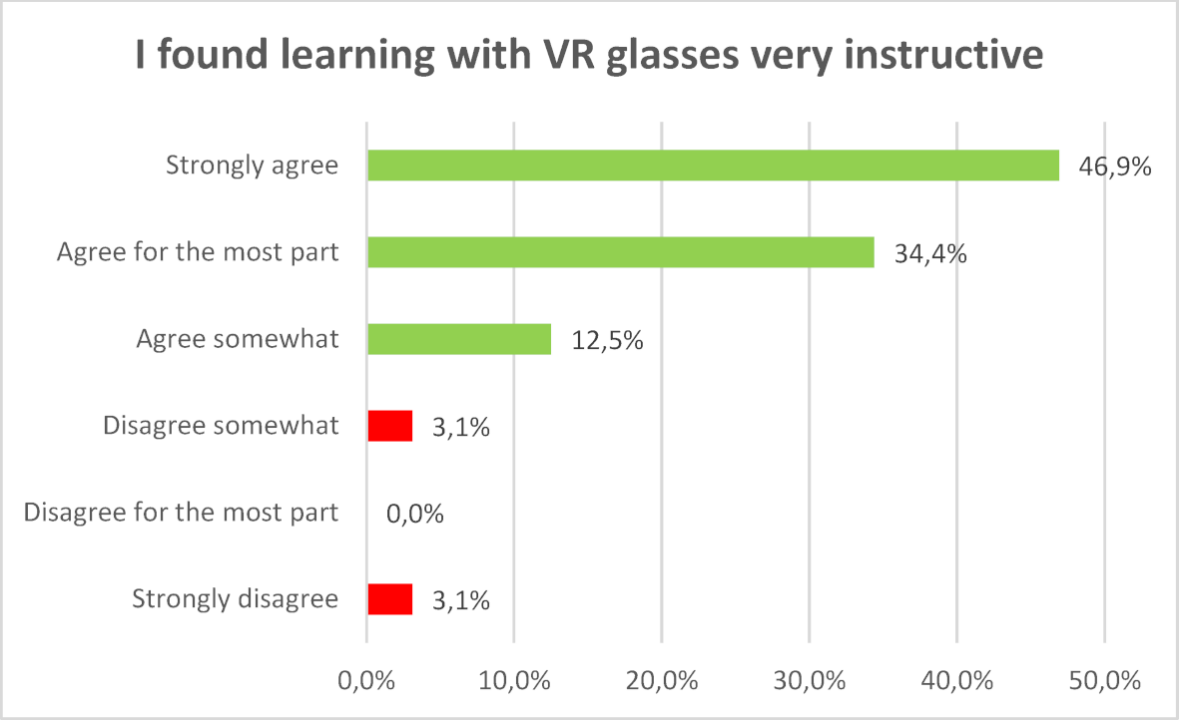
I found learning with VR glasses very instructive.

**Figure 2:**
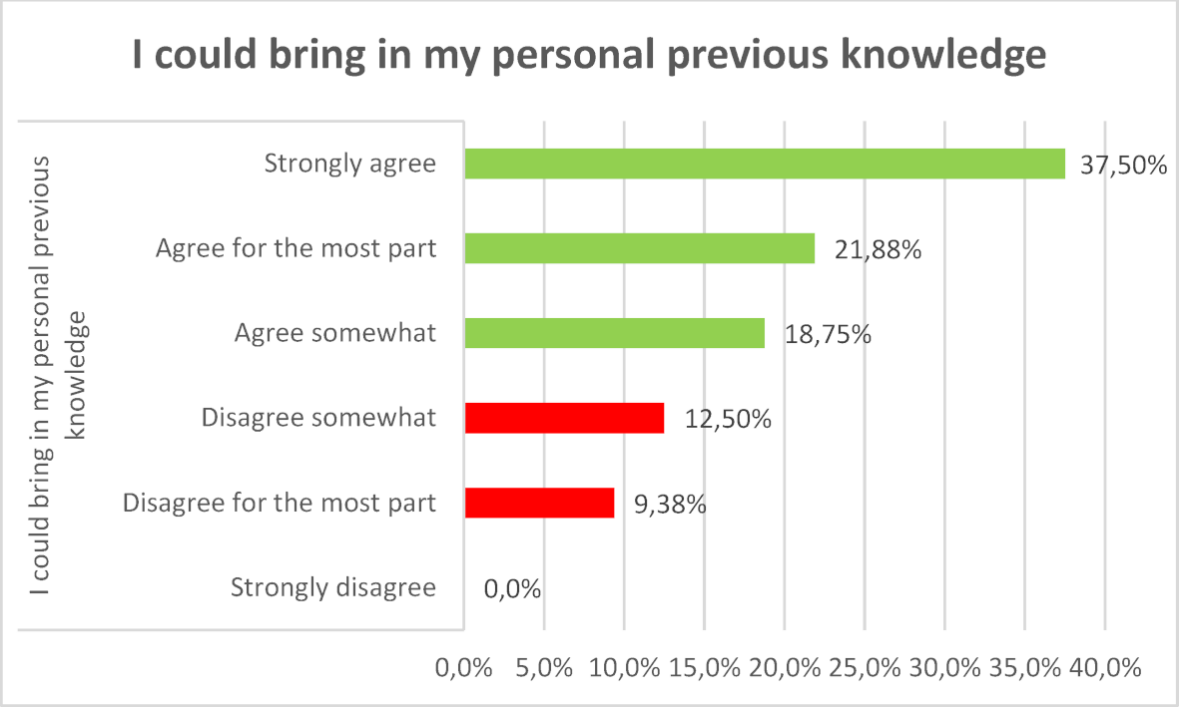
I could bring in my personal previous knowledge.

**Figure 3:**
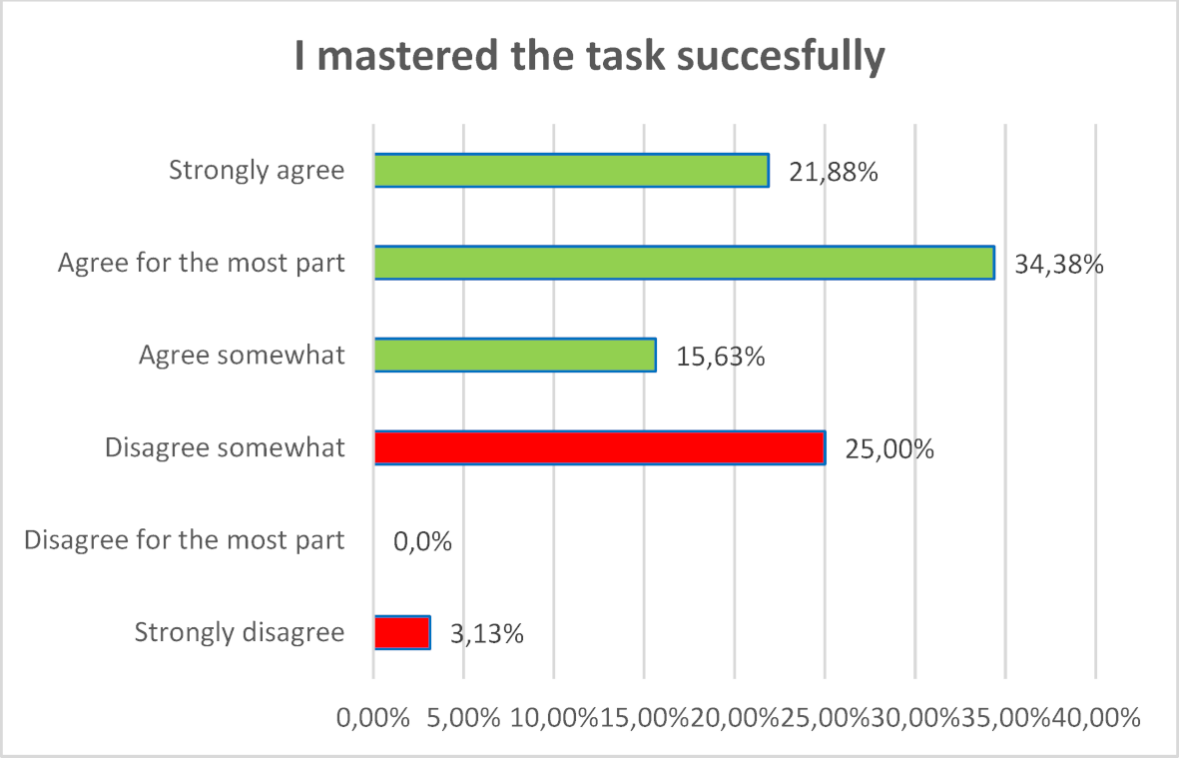
I mastered the task successfully.

For most participants the new learning technology did not lead to cognitive overload. They felt subjectively that they could build on their previous knowledge and learn something new. The survey showed that 80 percent of the participants largely or completely agreed that learning with VR glasses is instructive. The majority (72.34 percent) succeeded in solving the tasks (see figure 1 (Fig. 1), figure 2 (Fig. 2) and figure 3 (Fig. 3)).

#### Acceptance

**Figure 4:**
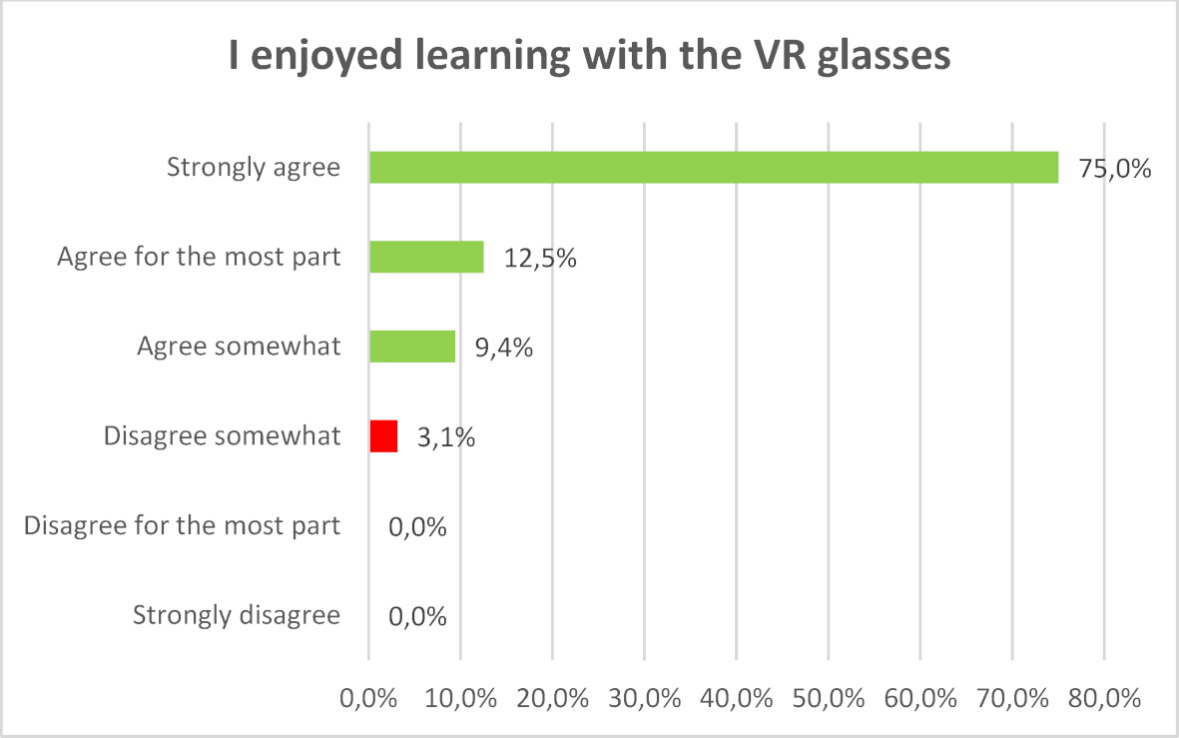
I enjoyed learning with the VR glasses.

75 percent of those surveyed say that learning with VR glasses is great fun. If you add the people who agreed “for the most part”, the figure rises to 86.5 percent (see figure 4 (Fig. 4)).

**Figure 5:**
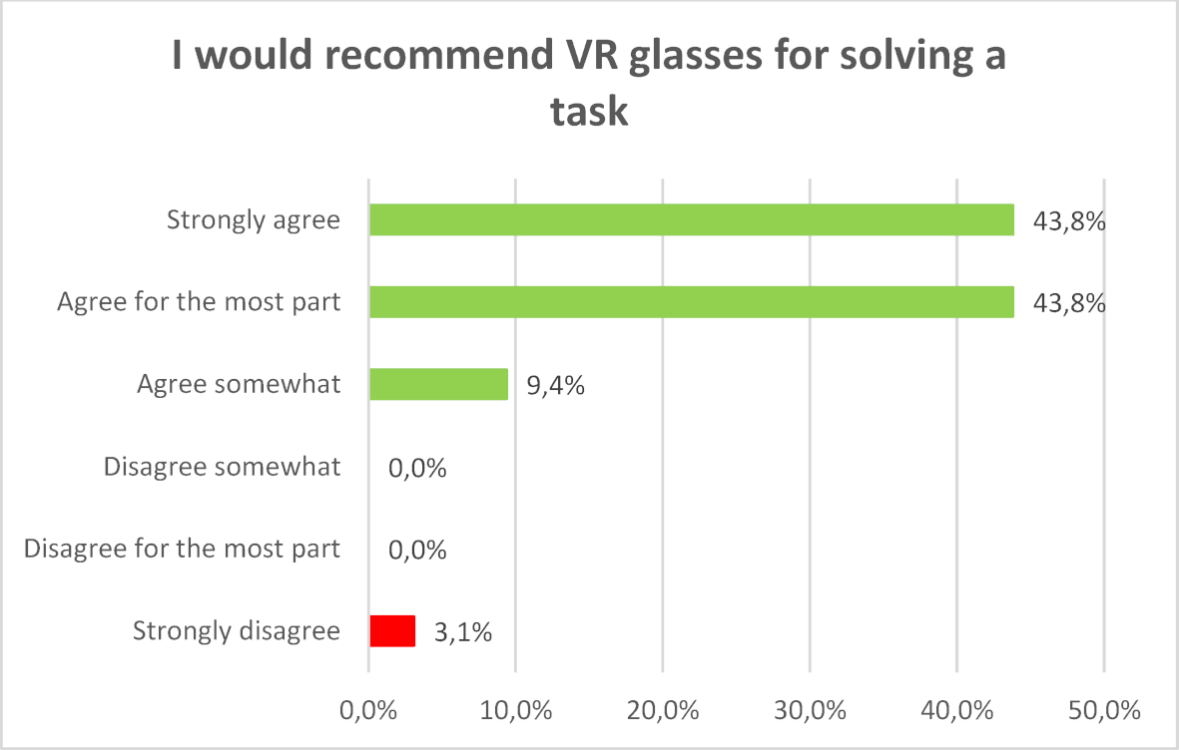
I would recommend VR glasses for solving a task.

Finally, 87.6 percent of the respondents would recommend learning with VR glasses whole-heartedly or for the most part (see figure 5 (Fig. 5)).

#### 3D-Visualisation/understandability

**Figure 6:**
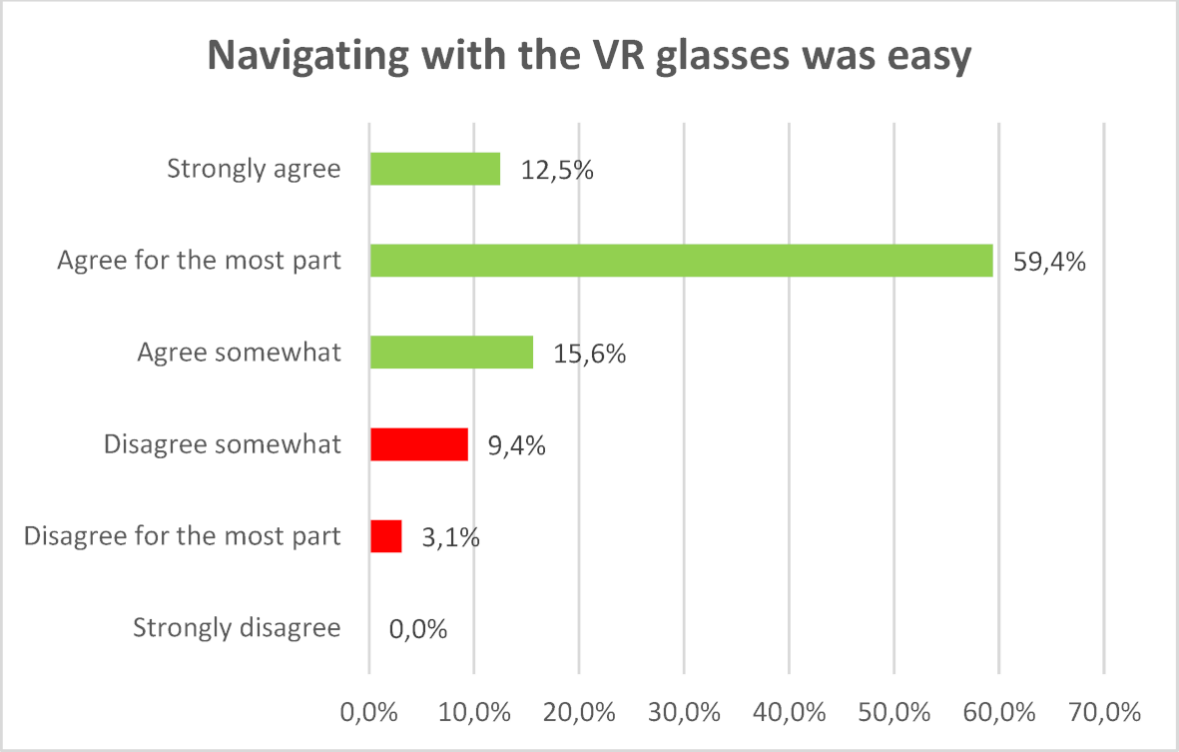
Navigating with the VR glasses was easy.

Although many of the respondents had little or no previous experience with VR glasses, they found them easy to handle for the most part (see figure 6 (Fig. 6)), despite the fact that the HTC Vive glasses and controllers used at the time of the intervention are considered today to be rather difficult to use.

**Figure 7:**
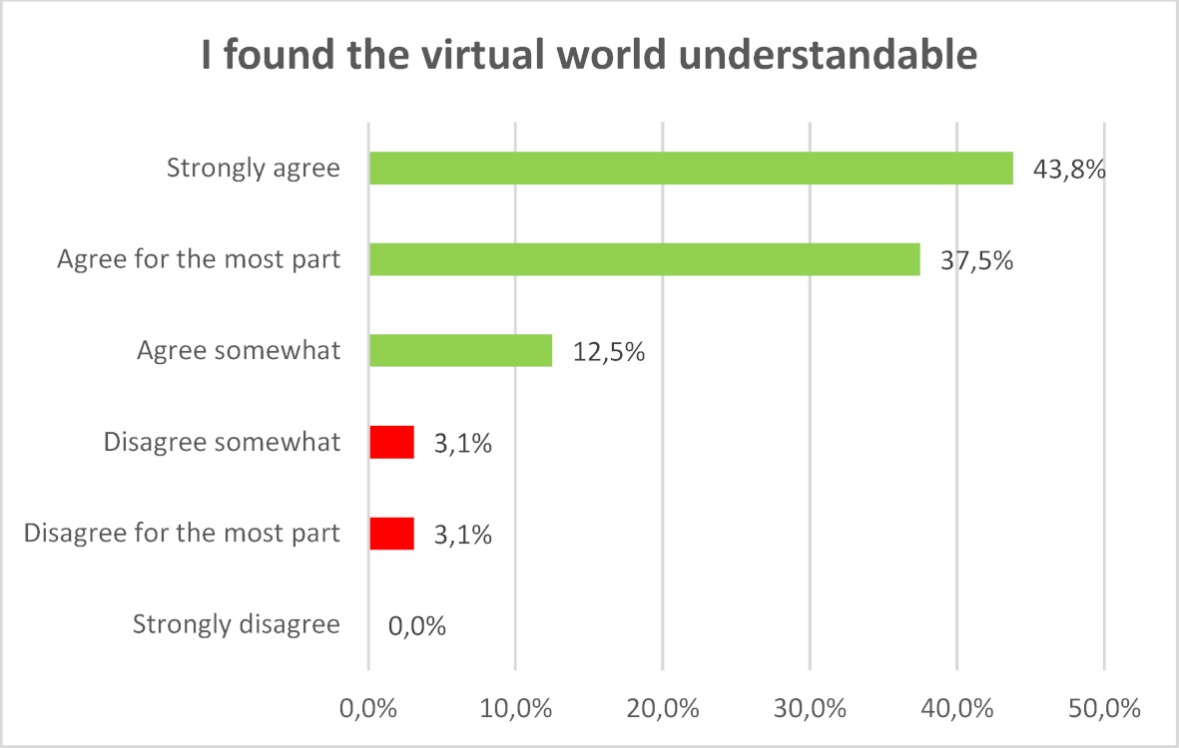
I found the virtual world understandable.

The systems for Virtual Reality as they are offered today are so far developed that even inexperienced users can find their way around the virtual world quite quickly and accomplish tasks. 88.2 percent of those surveyed said they found working with 3D visualisation easy all or most of the time (see figure 7 (Fig. 7)).

## Discussion

The current study has investigated the question whether the participants, a heterogeneous group of baby boomers and members of generations X and Y, perceive the new form of digital learning with VR glasses as cognitively stressful, whether they accept it, perceive it as understandable and experience it as conducive to their learning. The subject of the study is a pilot lesson on the anatomy of the heart using VR glasses.

Participants evaluated the course with VR glasses predominantly positively. Everyone seemed to find their way around the virtual reality relatively quickly. They were able to manage the setting. Even though the participants did not experience the VR glasses to be cognitively stressful, it must, however, be taken into account that not everyone perceives cognitive stress in the same way. According to Sweller [13] for example, older people, students and children experience different levels of cognitive stress under the same conditions, a fact which must be considered when planning a course with VR glasses. 

The participants had fun and from their point of view the use of VR glasses was beneficial for their learning. This may be due to the fact that in a virtual learning environment, knowledge need not be predetermined, but must be acquired exploratively. This explorative learning leads to an expansion of one`s personal space of experience [4]. According to Hofmann [14] the knowledge gained from these learning processes can be transferred to reality only if the built-in components are simulated and perceived as true to life as possible. Studies show that learning via simulations is more motivating and more conducive to learning than purely text-oriented forms of learning [15]. However, this way of learning does not result in a higher quality or quantity of cognitive processing and skill acquisition per se. Rather, without instruction, users feel more easily overwhelmed than with text-oriented forms of learning and lose the desire to learn with simulation. To avoid this, supporting measures are necessary: clear learning goals, work assignments and instructions, constantly available background information, as well as hints and exercises that encourage reflection [14]. These considerations were incorporated into the planning and implementation of the pilot project. The use of existing software and work assignments were systematically aligned with content and learning objectives. This careful selection of techniques and methods is absolutely crucial, so that the application of VR or new media and learning technologies can result in learning. As is often the case with new technologies, one could very easily become dangerously enthusiastic and lose sight of the real goal.

In the course we have described, the varying digital knowledge of the different generations is of little significance. Although commercially-operated virtual worlds are particularly popular with young people [https://www.slideshare.net/], this enthusiasm cannot be directly transferred to teaching. Teachers who plan a course using VR technology can refer to Kirschner et al. [16], who contradicts the generation concept and points out that all people must be treated equally, namely as cognitive learners. He is convinced that we should stop attributing special abilities to a specific group [16].

The simple study design without a control group, the small sample of 32 participants, the modified questionnaire and the lack of information regarding age on the data collection forms point to weaknesses of this study, as it is not possible to determine to which generations those participants belong whose assessment was rather negative. Even if the participating nurses had the feeling that learning with VR glasses was instructive, it remains unclear whether and how much they actually learned. Therefore, further studies with participants of different generations are necessary to make binding statements about increased learning with VR glasses. Many VR systems work without the need for a teaching staff.

## Conclusion

Nowadays, VR technology is no longer a challenge in terms of technical complexity [17] and it has provided learners easy access to the anatomy of the heart. Age-heterogeneous groups of learners should, therefore, not be an obstacle to integrating into a lesson new innovative complementary elements, such as those of VR technology. Our study shows that the majority of learners accept the use of VR glasses. They perceive the virtual world as understandable and beneficial for their learning. However, careful lesson preparation and optimal technical support are necessary to make the event a success. 

## Rights of use

The first author owns the copyrights to the work and the texts and graphics provided.

## Ethical consent

All participants were informed about the project and told that the survey was voluntary. Non-participation or the decision to halt participation bear no negative consequences. No financial resources were allocated for participation in the study.

## Competing interests

The authors declare that they have no competing interests.
